# The modification of individual factors on association between serum 25(OH)D and incident type 2 diabetes: Results from a prospective cohort study

**DOI:** 10.3389/fnut.2022.1077734

**Published:** 2022-12-29

**Authors:** Zhiyong Hu, Xueyuan Zhi, Yiming Ma, Jiafu Li, Jinxiu Wang, Jianliang Zhu, Bingyan Li, Zengli Zhang

**Affiliations:** ^1^School of Public Health and Management, Binzhou Medical University, Yantai, China; ^2^Department of Occupational and Environmental Health, School of Public Health, Suzhou Medical College of Soochow University, Suzhou, China; ^3^Jiangsu Key Laboratory of Preventive and Translational Medicine for Geriatric Diseases, School of Public Health, Suzhou Medical College of Soochow University, Suzhou, China; ^4^Lishui Center for Disease Control and Prevention, Lishui, China; ^5^Department of Nutrition and Food Hygiene, School of Public Health, Suzhou Medical College of Soochow University, Suzhou, China

**Keywords:** vitamin D, type 2 diabetes, physical activity, interaction, risk reclassification

## Abstract

Several epidemiological studies have suggested an association between low vitamin D status and increased risk for type 2 diabetes (T2D). This study aimed to explore the dose-response relationship of serum 25-hydroxyvitamin D [25(OH)D] concentrations with incident T2D and the interaction between serum 25(OH)D with individual factors on T2D risk. A total of 1,926 adults without diabetes (mean age: 52.08 ± 13.82 years; 42% men) were prospectively followed for 36 months. Cox proportional hazards model and restricted cubic spline analysis were performed to assess the association and dose-response relationship between serum 25(OH)D and T2D incidence. Both additive and multiplicative interactions were calculated between serum 25(OH)D and individual factors. The net reclassification index (NRI) was used to evaluate the improvement of risk prediction of T2D by adding serum 25(OH)D to traditional risk factors. There were 114 new T2D cases over a mean follow-up of 36 months. Serum 25(OH)D was not associated with T2D incidence, and no significant dose-response relationship was found in the total population. However, stratified analyses suggested a non-linear inverse relationship among individuals with baseline fasting plasma glucose (FPG) <5.6 mmol/L (*P*_overall_ = 0.061, *P*_non–linear_ = 0.048). And a significant multiplicative interaction was observed between serum 25(OH)D and FPG on T2D risk (*P* = 0.005). In addition, we found a significant additive interaction of low serum 25(OH)D with older age (RERI = 0.897, 95% CI: 0.080–1.714; AP = 0.468, 95% CI: 0.054–0.881), male (AP = 0.441, 95% CI: 0.010–0.871), and insufficient physical activity (RERI = 0.875, 95% CI: 0.204–1.545; AP = 0.575, 95% CI: 0.039–1.111) on T2D risk. Significant additive interactions were also observed between vitamin D deficiency/insufficiency with male, overweight/obesity, and insufficient physical activity on T2D risk. Moreover, adding low serum 25(OH)D to a model containing established risk factors yielded significant improvements in the risk reclassification of T2D (NRI = 0.205, 95% CI: 0.019–0.391). Our results indicated a non-linear relationship of serum 25(OH)D concentrations with T2D risk among individuals with normal FPG and additive interactions of serum 25(OH)D with gender, overweight/obesity, and physical activity on T2D risk, suggesting the importance of outdoor exercise.

## 1. Introduction

Type 2 diabetes (T2D) is a metabolic disease characterized by insulin resistance and pancreatic β-cell dysfunction. It was estimated that 537 million adults were living with diabetes in 2021, with an anticipated increase to 783 million by 2045 (1). The increasing incidence of T2D has prompted an urgent need for innovative methods to reverse this trend. Recently, vitamin D has attracted increasing public health interest because of its extra-skeletal effects (2, 3), including its association with the risk of developing T2D. It is biologically plausible that vitamin D may play a role in the pathogenesis of T2D, as insulin resistance and systematic inflammation have been reported with vitamin D deficiency *in vivo* and *in vitro* studies (4–6).

Several meta-analyses (7–9) summarized observational studies and suggested a negative association of blood 25-hydroxyvitamin D [25(OH)D] concentrations with T2D risk. However, the results of vitamin D supplementation for preventing T2D were inconsistent across intervention studies (2, 3, 10). For instance, the Diabetes Prevention with active Vitamin D (DPVD) randomized controlled trial indicated that active vitamin D treatment did not significantly decrease the incidence of diabetes among individuals with pre-diabetes (10). However, researchers did *post hoc* analyses and found that vitamin D supplementation significantly decreased the risk of diabetes in adults with a baseline body mass index (BMI) <30 kg/m^2^ (2). We have previously shown that gender and baseline BMI modified the effect of vitamin D supplementation on metabolic profile among patients with T2D (11). These results suggested that individual factors might modify the relationship between blood 25(OH)D concentrations and T2D risk.

Several previous studies investigated the modifying effects of BMI, gender, and intensive exercise (12–15). For instance, one study reported that baseline hypovitaminosis D_3_ successfully predicted hyperglycemia in the control group, but not in athletes, suggesting that physical activity might modify the association between vitamin D_3_ levels and metabolic risk (15). Nevertheless, few studies explored the potential additive interactions between these risk factors and serum 25(OH)D on T2D risk. It is possible that the joint effect of low vitamin D levels with established risk factors on T2D may exceed expectations based on the individual effect. Data from the National Health and Nutrition Examination Survey (NHANES) indicated that the interaction of insufficient 25(OH)D and high BMI explained 47% of the increased odds of insulin resistance (16). Since vitamin D deficiency and insufficiency are easy to be diagnosed and cured, exploring the additive interactions of serum 25(OH)D with established risk factors on T2D risk may be helpful to identify the susceptible population who can be greatly benefited from improving vitamin D status.

Moreover, the changes in biological effects as serum 25(OH)D increases are S-shaped rather than linear (17). Several studies investigated the dose-response relationship between serum 25(OH)D and T2D risk and yielded inconsistent results (18–21). However, most previous studies were conducted in Europe where vitamin D supplements are commonly used, and the dose-response relationship of serum 25(OH)D with T2D risk has not been studied among the Chinese population.

In addition, previous studies reported that adding serum 25(OH)D to the Framingham Risk Score could improve coronary heart disease risk prediction in patients with essential hypertension or patients with T2D (22, 23). However, little was known about whether baseline serum 25(OH)D could refine risk prediction for T2D.

Thus, we conducted a prospective cohort study in Chinese adults to assess the dose-response relationship between serum 25(OH)D concentrations and the risk of T2D, and to explore the interactions of serum 25(OH)D with established risk factors on T2D risk. We also investigated whether serum 25(OH)D might improve the predictive value of T2D beyond conventional risk factors.

## 2. Materials and methods

### 2.1. The ethics board approval

The current study conformed to the principles set by the Declaration of Helsinki and was approved by the Ethics Committee of Soochow University (ESCU-20160001). Written informed consent was obtained from all participants included in the study.

### 2.2. Study population

From 1 September to 14 October 2013, a total of 2,072 adults from the Liandu district in Lishui city, in east-central China, of Han ethnicity were invited to participate in the study. Liandu district consists of 16 blocks, and approximately 400,000 people live there. A multistage sampling method was applied to the present study. First, 4 residence communities in each block were sampled by simple random sampling. Then, systematic sampling was adopted to select 33 households in each residence community. Finally, one adult who had lived in the Liandu district for at least 2 years, was randomly selected from each household without replacement. In total, 2,072 residents were enrolled. Of these invited residents, 1,926 individuals met the inclusion criteria for the study: without diabetes, without malignancy, without chronic liver or renal diseases, and without using vitamin D supplementation (based on a self-assessment questionnaire). A follow-up survey was conducted for all participants in October 2016 to collect data on fasting plasma glucose (FPG) and the development of T2D.

### 2.3. Primary endpoint

The development of T2D was the primary endpoint in this study. New-onset T2D was defined as a self-reported history of a physician diagnosis during the follow-up period, and/or receiving pharmacological treatment for T2D, and/or an FPG ≥7.0 mmol/L (24).

### 2.4. Baseline measurements

At the baseline visit, standardized questionnaires were used to collect information regarding participants’ demographics [i.e., age, gender, and residential district (rural or urban)], socio-economic status (including education level), smoking status, alcohol consumption, physical activity, and family history of diabetes]. Education level was categorized into four groups: no school, primary school, middle school, and junior college or higher. For smoking status, the research population was categorized into three groups: current smokers (smoking during the last 12 months or quit smoking less than 6 months ago), former smokers (quit smoking more than 6 months ago), and non-smokers (never smoked). For alcohol consumption, the research population was categorized as current drinkers (at least one time per week), former drinkers (quit drinking more than 6 months ago), and non-drinkers (never drank). According to the WHO recommendations, sufficient physical activity was defined as engaging in at least 150 min of moderate-intensity activity per week or equivalent (25). Family history was judged as positive when at least one of the first-degree relatives had diabetes.

Height (in cm) was measured with a wall-mounted stadiometer and weight (in kg) was measured with a balance-beam scale. BMI was calculated as body weight divided by height squared (kg/m^2^). BMI ≥28.0, 24.0–27.9, and <24.0 kg/m^2^ was classified as obesity, overweight, and normal weight (26). Blood pressure was measured three times while participants were in the relaxed sitting position after 15 min of rest. There was a 5-min rest period between each measurement and the mean value of the three measurements was used for analysis. Hypertension was defined as mean systolic blood pressure (SBP) ≥140 mmHg and/or diastolic blood pressure (DBP) ≥90 mmHg and/or the use of antihypertensive medications.

### 2.5. Data collection and definitions

Participants were instructed to take all regular medications, but not aspirin or non-steroidal anti-inflammatory drugs for 48 h before the visit. Participants were further requested to refrain from smoking for 1 h and from vigorous physical activity for 12 h before the visit. A venous blood sample was collected from each participant following an 8-h overnight fast for biochemical analysis. Aliquots of serum, plasma, and buffy coat were frozen and shipped on dry ice to Lishui Center for Disease Control and Prevention and stored at −80°C for future assays. Serum 25(OH)D concentration was determined using an automatic chemiluminescence immunoassay analyzer (ADVIA Centaur XP, Siemens Healthcare Diagnostics Inc., Tarrytown, NY, USA). According to the Endocrine Society clinical practice guideline (27), vitamin D deficiency, insufficiency, and sufficiency were defined as serum 25(OH)D <20, 20–29.9, and ≥30 ng/ml. FPG concentration was determined by the hexokinase method using an automatic biochemical analyzer (COBAS c702, Roche Diagnostics Gmbh, Mannheim, Germany). Based on one of the diagnostic criteria for prediabetes proposed by the American Diabetes Association (24), impaired fasting glucose (IFG) was defined as 5.6 mmol/L ≤ FPG < 7.0 mmol/L. The concentrations of triglyceride (TG), total cholesterol (TC), high-density lipoprotein cholesterol (HDL-C), and low-density lipoprotein cholesterol (LDL-C) were measured by an enzymatic method on the automatic biochemistry analyzer (COBAS c702). Dyslipidemia was defined as TC ≥6.2 mmol/L and/or TG ≥2.3 mmol/L and/or HDL-C <1.0 mmol/L according to Chinese guideline for the management of dyslipidemia in adults (28).

### 2.6. Statistical analysis

All data were analyzed by using SAS version 9.4 (SAS Institute, Cary, NC, USA) and STATA version 16 (StataCorp, TX, USA), and statistical significance was defined as a two-sided *P*-value < 0.05. Data were presented as mean ± standard deviation or median (interquartile range: IQR) for continuous variables and as *n* (%) for categorical variables. Participants were divided into two groups using the median of serum 25(OH)D. The differences in quantitative variables between the two groups were compared by using independent Student’s *t*-tests or Wilcoxon test as appropriate. Qualitative variables were tested by Chi-square analysis. The Cox proportional hazard regression model was performed to determine the hazard ratio (HR) for the risk of developing T2D and serum 25(OH)D concentration with T2D incidence as the dependent variable. Serum 25(OH)D was analyzed as a continuous variable or categorical variable which was categorized using median, thresholds of vitamin D deficiency and insufficiency, quartiles, and quintiles. In model 1, only serum 25(OH)D was included as the independent variable. Then age, gender, and BMI were included in model 2. In model 3, the following variables were added to model 2: residential district, education level, FPG, hypertension, dyslipidemia, smoking status, alcohol use, physical activity, and family history of diabetes. Tests for trends were performed by entering the median value of each category of serum 25(OH)D as a continuous variable in the models. We further calculated the HRs for serum 25(OH)D and T2D across strata of age, gender, residential district, BMI, physical activity, and baseline FPG. To further explore the possible dose-response or non-linear relationship between serum 25(OH)D concentrations and T2D risk, we used a restricted cubic spline model with three knots located at 10, 20, and 35 ng/ml (the reference: 20 ng/ml) (29). Both additive and multiplicative interactions between serum 25(OH)D (categorized using median or the threshold of vitamin D deficiency/insufficiency) and some established risk factors on the risk of T2D were calculated. Additive interaction better reflects the presence of biological interaction (30). To evaluate additive interaction, the relative excess risk due to interaction (RERI), attributable proportion of interaction (AP), and synergy index (S) were calculated as follows: RERI = HR_11_-HR_10_-HR_01_ + 1, AP = RERI/HR_11_, S = (HR_11_-1)/((HR_10_-1) + (HR_01_-1)), where the subscripts indicate the presence (1) or absence (0) of the two risk factors (31). In the absence of additive interaction, RERI and AP are equal to 0, and S is equal to 1. A cross-product interaction term was set in the Cox proportional hazard regression model to assess multiplicative interaction. The improvement of T2D risk prediction by adding serum 25(OH)D (categorized using median) to traditional risk factors (variables adjusted in model 3) was assessed using the net reclassification index (NRI) and integrated discrimination improvement (IDI) (32, 33). The calibration of each model was evaluated by using Gronnesby and Borgan χ^2^ statistic. Akaike and Bayesian information criteria were calculated to assess the global goodness of fit for each model. Likelihood ratio test was used to compare the global fit of the two models. The discrimination power of each model was evaluated by using Harrell’s C index.

## 3. Results

### 3.1. Baseline characteristics of the study population

Table 1 summarized the baseline characteristics of the study populations. The mean age of the study population was 52.08 ± 13.82 years, and 808 (41.95%) were men. The median serum 25(OH)D concentration was 25.415 ng/ml (IQR: 21.14–29.55 ng/ml). Of 1,926 participants, percentage of vitamin D deficiency (<20 ng/ml) and insufficiency (20–29.9 ng/ml) was 19.37 and 57.84%, respectively.

**TABLE 1:** Baseline characteristics of the study population according to serum 25(OH)D concentrations (*n* = 1,926).

Characteristic	Total (*n* = 1,926)	Serum 25(OH)D concentrations (ng/ml)		*P*
		**<25.415 (*n* = 963)**	**≥25.415 (*n* = 963)**	
25(OH)D, median (IQR)	25.415 (21.14–29.55)	21.14 (17.93–23.55)	29.55 (27.37–32.45)	
Age (years)	52.08 ± 13.82	51.65 ± 14.18	52.52 ± 13.44	0.164
Sex (men), *n* (%)	808 (41.95)	294 (30.53)	514 (53.37)	<0.001
District (rural), *n* (%)	754 (39.15)	328 (34.06)	426 (44.24)	<0.001
Education, *n* (%)				0.118
No school	327 (16.98)	164 (16.93)	163 (17.03)	
Primary school	524 (27.21)	256 (26.58)	268 (27.83)	
Middle school	938 (48.70)	461 (47.87)	477 (49.53)	
Junior college or higher	137 (7.11)	82 (8.52)	55 (5.71)	
Body mass index (kg/m^2^)	23.73 ± 3.32	23.94 ± 3.43	23.53 ± 3.18	0.007
Normal (<24)	1,091 (56.65)	516 (53.58)	575 (59.71)	0.009
Overweight (24–28)	640 (33.23)	334 (34.68)	306 (31.78)	
Obesity (≥28)	195 (10.12)	113 (11.73)	82 (8.52)	
FPG (mmol/L)	5.65 ± 0.59	5.69 ± 0.58	5.61 ± 0.60	0.003
IFG, *n* (%)	996 (51.71)	516 (53.58)	480 (49.84)	0.101
Hypertension, *n* (%)	632 (32.81)	318 (33.02)	314 (32.61)	0.846
Dyslipidemia, *n* (%)	832 (43.20)	471 (48.91)	361 (37.49)	<0.001
Current smoker, *n* (%)	415 (21.55)	168 (17.45)	247 (25.65)	<0.001
Current drinker, *n* (%)	585 (30.37)	225 (23.36)	360 (37.38)	<0.001
Sufficient physical activity, *n* (%)	429 (22.27)	235 (24.40)	194 (20.15)	0.025
Family history of diabetes, *n* (%)	54 (2.8)	35 (3.63)	19 (1.97)	0.027

IQR, interquartile range; FPG, fasting plasma glucose; IFG, impaired fasting glucose.

As shown in Table 1, participants were divided into two groups according to the median of serum 25(OH)D. Subjects with serum 25(OH)D ≥25.415 ng/ml tended to be men, current smokers, current drinkers, live in rural areas, and have lower BMI and FPG (all *P*-values < 0.01), but were less likely to have dyslipidemia, sufficient physical activity, and family history of diabetes (all *P*-values < 0.05). There was no significant difference in age, education level, proportions of IFG, and hypertension between the two groups.

### 3.2. Associations of serum 25(OH)D concentrations with T2D incidence

As shown in Table 2, during the 36 months of follow-up, 114 participants (5.9%) developed incident T2D. Taking subjects with serum 25(OH)D ≥25.415 ng/ml as reference, those with 25(OH)D <25.415 ng/ml did have an increased risk of incident T2D in model 1 (HR = 1.48, 95% CI: 1.02–2.16; *P* = 0.039) and model 2 (adjusted HR = 1.49, 95% CI: 1.02–2.20; *P* = 0.042). However, after further adjustments for potential confounders, this association did not reach statistical significance in model 3 (adjusted HR = 1.36, 95% CI: 0.92–1.99; *P* = 0.120). Compared with vitamin D-sufficient participants, those with vitamin D deficiency and insufficiency had a higher risk of developing T2D in model 1 and model 2. But these associations were not significant in model 3. The HRs (95% CIs) were 1.83 (0.96–3.47) and 1.60 (0.92–2.79) for vitamin D deficient and insufficient subjects. No significant linear trend was observed in model 3 (*P* = 0.076). When considered as a continuous variable, a 10-ng/ml higher 25(OH)D concentration was not significantly associated with T2D incidence in models 1–3 (Table 2). When categorized using quartiles and quintiles, serum 25(OH)D concentrations was not significantly associated with T2D incidence in model 3 (Table 2).

**TABLE 2:** Associations of serum 25(OH)D concentrations with incident type 2 diabetes.

25(OH)D (ng/ml)	Events (%)	Model 1		Model 2		Model 3	
		**HR (95% CI)**	***P***	**HR (95% CI)**	***P***	**HR (95% CI)**	***P***
High (≥25.415)	46 (4.78)	1.00	–	1.00	–	1.00	
Low (<25.415)	68 (7.06)	1.48 (1.02–2.16)	0.039	1.49 (1.02–2.20)	0.042	1.36 (0.92–1.99)	0.120
Without vitamin D deficiency (≥20)	88 (5.67)	1.00	–	1.00	–	1.00	–
Vitamin D deficiency (<20)	26 (6.97)	1.23 (0.79–1.90)	0.356	1.18 (0.75–1.84)	0.470	1.26 (0.80–1.97)	0.319
Vitamin D sufficiency (≥30)	16 (3.64)	1.00	–	1.00	–	1.00	–
Vitamin D deficiency or insufficiency (<30)	98 (6.59)	1.82 (1.07–3.09)	0.026	1.83 (1.07–3.14)	0.028	1.66 (0.96–2.84)	0.068
Vitamin D sufficiency (≥30)	16 (3.64)	1.00	–	1.00	–	1.00	–
Vitamin D insufficiency (20–29.9)	72 (6.46)	1.79 (1.04–3.07)	0.036	1.81 (1.05–3.14)	0.034	1.60 (0.92–2.79)	0.094
Vitamin D deficiency (<20)	26 (6.97)	1.92 (1.03–3.58)	0.040	1.89 (1.00–3.60)	0.051	1.83 (0.96–3.47)	0.065
*P* for trend*		0.049		0.068		0.076	
Q4 (≥29.55)	20 (4.15)	1.00	–	1.00	–	1.00	–
Q3 (25.415–29.55)	26 (5.41)	1.30 (0.73–2.33)	0.379	1.30 (0.72–2.33)	0.381	1.23 (0.68–2.22)	0.499
Q2 (21.14–25.415)	36 (7.47)	1.81 (1.05–3.13)	0.034	1.85 (1.06–3.22)	0.030	1.56 (0.89–2.72)	0.121
Q1 (<21.14)	32 (6.65)	1.60 (0.92–2.80)	0.098	1.59 (0.89–2.83)	0.117	1.48 (0.83–2.63)	0.185
*P* for trend*		0.064		0.079		0.146	
Q5 (≥30.61)	16 (4.12)	1.00	–	1.00	–	1.00	–
Q4 (26.92–30.61)	19 (4.96)	1.20 (0.62–2.34)	0.587	1.23 (0.63–2.40)	0.544	1.10 (0.56–2.16)	0.781
Q3 (23.98–26.92)	22 (5.71)	1.39 (0.73–2.65)	0.314	1.44 (0.75–2.75)	0.273	1.23 (0.64–2.36)	0.539
Q2 (20.17–23.98)	30 (7.79)	1.91 (1.04–3.50)	0.037	1.92 (1.03–3.58)	0.040	1.55 (0.83–2.90)	0.172
Q1 (<20.17)	27 (7.01)	1.70 (0.92–3.16)	0.091	1.71 (0.90–3.24)	0.099	1.59 (0.84–3.01)	0.152
*P* for trend*		0.033		0.045		0.078	
Continuous^#^	114 (5.92)	0.75 (0.56–1.00)	0.052	0.76 (0.57–1.03)	0.072	0.80(0.59–1.09)	0.155

Model 1: unadjusted; model 2: adjusted for age, gender, and body mass index; model 3: adjusted for model 2 and district, education level, fasting plasma glucose, hypertension, dyslipidemia, smoking status, alcohol drinking, physical activity, and family history of diabetes. HR, hazard ratio; CI, confidence interval.

*Test for trend based on the variable containing the median value for each group.

^#^HR was scaled to 10-ng/ml higher serum 25(OH)D concentrations.

### 3.3. The dose-response relationship between serum 25(OH)D concentrations with T2D risk

**FIGURE 1:**
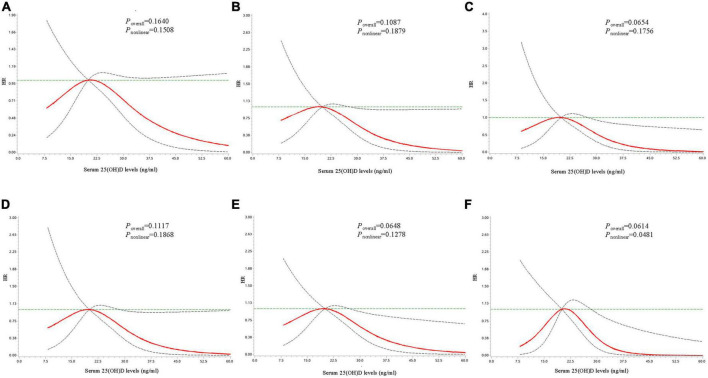
Dose-response curves for the associations between serum 25(OH)D concentrations with risk of developing type 2 diabetes. Dose-response curves for the associations between serum 25(OH)D with risk of developing type 2 diabetes in the total population **(A)**, among individuals with age ≥52 years **(B)**, with male gender **(C)**, with overweight/obesity **(D)**, with insufficient physical activity **(E)**, and with fasting plasma glucose <5.6 mmol/L at baseline **(F)**. The solid line and dashed line represent the adjusted estimated HRs and 95% confidence intervals. Analyses were adjusted for age, gender, body mass index, district, education level, fasting plasma glucose, hypertension, dyslipidemia, smoking status, alcohol drinking, physical activity, and family history of diabetes.

Restricted cubic spline analyses were conducted to explore the possible dose-response or non-linear relationship of serum 25(OH)D concentrations with the risk of T2D. As shown in Figure 1, no significant dose-response relationship between serum 25(OH)D and T2D risk was observed among the total population (*P*_overall_ = 0.1640, *P*_non–linear_ = 0.1508). Stratified analysis suggested a non-linear inverse relationship among individuals with baseline FPG <5.6 mmol/L (*P*_overall_ = 0.061, *P*_non–linear_ = 0.048; Figure 1). When the serum 25(OH)D concentrations was between approximately 28.5 and 40 ng/ml, HR decreased rapidly with the increase of serum 25(OH)D (Supplementary Table 1). And when the serum 25(OH)D concentrations reached or exceeded approximately 40 ng/ml, the reducing trend of HR value tended to be flat. For participants with baseline FPG <5.6 mmol/L, the adjusted HRs of T2D were 0.38 (95% CI: 0.16–0.92) at 30 ng/ml, 0.05 (95% CI: 0.00–0.60) at 40 ng/ml, 0.01 (95% CI: 0.00–0.42) at 50 ng/ml compared with 20 ng/ml (Table 3). No significant dose-response relationship between serum 25(OH)D concentrations and T2D risk was observed among other subgroups (Supplementary Figures 1, 2).

**TABLE 3:** Hazard ratios (95% CIs) of type 2 diabetes by serum 25(OH)D concentrations.

	HR (95% CI)*			
	**20 ng/ml**	**30 ng/ml**	**40 ng/ml**	**50 ng/ml**
Total	1.00 (reference)	0.73 (0.50–1.05)	0.37 (0.14–1.03)	0.19 (0.03–1.06)
**Age**				
<52 years	1.00 (reference)	1.14 (0.60–2.16)	0.82 (0.16–4.23)	0.58 (0.03–9.76)
≥52 years	1.00 (reference)	0.60 (0.37–0.97)	0.25 (0.06–0.94)	0.10 (0.01–0.94)
**Gender**				
Male	1.00 (reference)	0.53 (0.30–0.92)	0.17 (0.04–0.77)	0.05 (0.00–0.70)
Female	1.00 (reference)	1.02 (0.62–1.67)	0.89 (0.22–3.54)	–
**District**				
Rural	1.00 (reference)	0.71 (0.36–1.42)	0.29 (0.05–1.82)	0.11 (0.00–2.62)
Urban	1.00 (reference)	0.77 (0.50–1.20)	0.46 (0.14–1.56)	0.27 (0.04–2.14)
**BMI**				
<24 kg/m^2^	1.00 (reference)	0.95 (0.55–1.63)	0.79 (0.20–3.19)	0.65 (0.06–6.79)
≥24 kg/m^2^	1.00 (reference)	0.58 (0.34–0.98)	0.21 (0.05–0.94)	0.13 (0.02–0.94)
**Physical activity**				
Sufficient	1.00 (reference)	1.40 (0.58–3.35)	1.79 (0.18–17.80)	–
Insufficient	1.00 (reference)	0.61 (0.40–0.93)	0.25 (0.08–0.81)	0.10 (0.01–0.73)
**FPG**				
<5.6 mmol/L	1.00 (reference)	0.38 (0.16–0.92)	0.05 (0.00–0.60)	0.01 (0.00–0.42)
≥5.6 mmol/L	1.00 (reference)	0.92 (0.61–1.38)	0.77 (0.25–2.37)	0.64 (0.10–4.32)

BMI, body mass index; FPG, fasting plasma glucose; HR, hazard ratio; CI, confidence interval.

*Adjusted for age, gender, body mass index, district, education level, fasting plasma glucose, hypertension, dyslipidemia, smoking status, alcohol drinking, physical activity, and family history of diabetes.

The symbol “–”indicates the maximum value of serum 25(OH)D concentrations in these two subgroups were less than 50 ng/ml.

For individuals with baseline FPG <5.6 mmol/L, those in the second quartile had a higher risk of T2D than those in the highest quartile (adjusted HR = 8.38, 95% CI: 1.82–38.60; Supplementary Table 2), and those in the second (adjusted HR = 5.96, 95% CI: 1.22–29.03) and third (adjusted HR = 5.14, 95% CI: 1.06–25.08) quintile had an increased T2D risk than those in the highest quintile (Supplementary Table 3).

### 3.4. Interaction analysis of serum 25(OH)D with conventional risk factors on T2D risk

Participants with serum 25(OH)D concentrations less than median (25.415 ng/ml) were classified as low 25(OH)D group, and others were defined as high 25(OH)D group. As shown in Table 4, stratified analyses indicated that the significant relationship of low 25(OH)D with increased risk of T2D was limited to men (adjusted HR = 1.82, 95% CI: 1.03–3.21; *P* = 0.039), individuals with overweight/obesity (adjusted HR = 1.64, 95% CI: 1.00–2.68; *P* = 0.048), insufficient physical activity (adjusted HR = 1.58, 95% CI: 1.03–2.42; *P* = 0.037), FPG <5.6 mmol/L at baseline (adjusted HR = 2.78, 95% CI: 1.18–6.51; *P* = 0.019). The RERI and AP for the interaction of low serum 25(OH)D with older age was 0.651 (−0.095 to 1.398) and 0.464 (−0.050 to 0.977), while S was not statistically significant, suggesting a potential additive interaction between these two factors on T2D risk. And about 46.4% of the HR of being T2D was attributable to the interaction between these two risk factors. The AP for the interaction of low serum 25(OH)D with male was 0.441 (95% CI: 0.01–0.871), while RERI and S were not statistically significant (Table 4), suggesting a potential additive interaction between these two factors. For additive interaction between low serum 25(OH)D and insufficient physical activity (Table 4), the estimated RERI and AP were 0.875 (95% CI: 0.204–1.545) and 0.575 (95% CI: 0.039–1.111), respectively, while S was not statistically significant, suggesting that there might be a potential additive interaction between low serum 25(OH)D and insufficient physical activity on T2D risk. A significant multiplicative interaction effect was observed between low serum 25(OH)D and FPG on T2D risk (*P* = 0.005).

**TABLE 4:** Interactions between serum 25(OH)D and established risk factors on type 2 diabetes risk.

	Events (%)	HR (95% CI)*	*P* *	RERI (95% CI)	AP (95% CI)	S (95% CI)	*P*_interaction_ ^#^
Total	114 (5.92)	1.36 (0.92–1.99)	0.120				
Age				0.897 (0.080 to 1.714)	0.468 (0.054 to 0.881)	42.688 (0 to NA)^b^	0.138
<52 years	38 (3.93)	0.95 (0.49–1.84)	0.870				
≥52 years	76 (7.92)	1.56 (0.96–2.53)	0.074				
Gender				0.766 (−0.064 to 1.596)	0.441 (0.010 to 0.871)^a^	−26.391 (NA)^b^	0.134
Male	53 (6.56)	1.82 (1.03–3.21)	0.039				
Female	61 (5.46)	1.04 (0.62–1.76)	0.877				
District				0.042 (−1.003 to 1.088)	0.024 (−0.571 to 0.619)	1.059 (0.245 to 4.574)	0.908
Rural	36 (4.77)	1.32 (0.65–2.67)	0.446				
Urban	78 (6.66)	1.34 (0.84–2.14)	0.219				
BMI				0.943 (−0.120 to 2.005)	0.372 (−0.021 to 0.764)	2.585 (0.415 to 16.117)	0.114
<24 kg/m^2^	40 (3.67)	1.10 (0.57–2.13)	0.784				
≥24 kg/m^2^	74 (8.86)	1.64 (1.00–2.68)	0.048				
Physical activity				0.875 (0.204–1.545)^a^	0.575 (0.039 to 1.111)^a^	−1.471 (NA)^b^	0.087
Sufficient	21 (4.90)	0.79 (0.31–2.07)	0.637				
Insufficient	93 (6.21)	1.58 (1.03–2.42)	0.037				
FPG				−1.729 (−4.031 to 0.574)	−1.643 (−3.442 to 0.156)	0.029 (0–1.08 × 10^6^)	0.005
<5.6 mmol/L	29 (3.12)	2.78 (1.18–6.51)	0.019				
≥5.6 mmol/L	85 (8.53)	1.07 (0.69–1.66)	0.763				

BMI, body mass index; FPG, fasting plasma glucose; HR, hazard ratio; CI, confidence interval; RERI, relative excessive risk due to interaction; AP, attributable proportion due to interaction; S, the synergy index.

*Serum 25(OH)D was categorized using median (25.415 ng/ml) and the reference group was serum 25(OH)D ≥25.415 ng/ml, the HRs were adjusted for age, gender, body mass index, district, education level, fasting plasma glucose, hypertension, dyslipidemia, smoking status, alcohol drinking, physical activity, and family history of diabetes.

^#^*P* for multiplicative interaction.

^a^Statistically significant with RERI >0 and AP >0 indicating additive interaction.

^b^The 95% CI of S was not available because the values of S were less than 0.

When stratified by the threshold of vitamin D deficiency/insufficiency [serum 25(OH)D <30 ng/ml], a significant association of vitamin D deficiency/insufficiency with increased risk of T2D was found among men (adjusted HR = 2.26, 95% CI: 1.05–4.86; *P* = 0.037), individuals with overweight/obesity (adjusted HR = 2.42, 95% CI: 1.04–5.63; *P* = 0.040), individuals with insufficient physical activity (adjusted HR = 1.92, 95% CI: 1.03–3.57; *P* = 0.039), and individuals with FPG <5.6 mmol/L at baseline (adjusted HR = 4.93, 95% CI: 1.14–21.35; *P* = 0.033; Table 5). And the RERI for the interactions of vitamin D deficiency/insufficiency with male, overweight/obesity, and insufficient physical activity were 0.857 (95% CI: 0.104–1.609), 1.382 (95% CI: 0.294–2.470), and 0.877 (95% CI: 0.200–1.554), respectively, while the corresponding AP and S were not statistically significant, except the AP for the interaction of vitamin D deficiency/insufficiency with overweight/obesity (0.570, 95% CI: 0.116–1.024; Table 5).

**TABLE 5:** Interactions between vitamin D deficiency/insufficiency and established risk factors on type 2 diabetes risk.

	HR (95% CI)*	*P* *	RERI (95% CI)	AP (95% CI)	S (95% CI)	*P*_interaction_ ^#^
Total	1.66 (0.96–2.84)	0.068				
Age			0.686 (−0.466 to 1.839)	0.284 (−0.271 to 0.838)	1.937 (0.210 to 17.838)	0.541
<52 years	1.66 (0.63–4.40)	0.305				
≥52 years	1.61 (0.83–3.11)	0.161				
Gender			0.857 (0.104 to 1.609)^a^	0.535 (−0.037 to 1.108)	−2.346 (NA)^b^	0.144
Male	2.26 (1.05–4.86)	0.037				
Female	1.09 (0.51–2.30)	0.826				
District			0.212 (−1.062 to 1.486)	0.101 (−0.538 to 0.739)	1.237 (0.251 to 6.089)	0.969
Rural	1.57 (0.63–3.91)	0.336				
Urban	1.63 (0.83–3.21)	0.160				
BMI			1.382 (0.294 to 2.470)^a^	0.570 (0.116 to 1.024)^a^	33.084 (0 to NA)^b^	0.112
<24 kg/m^2^	0.98 (0.46–2.13)	0.967				
≥24 kg/m^2^	2.42 (1.04–5.63)	0.040				
Physical activity			0.877 (0.200 to 1.554)^a^	0.605 (−0.189 to 1.399)	−1.056 (NA)^b^	0.181
Sufficient	0.88 (0.26–3.01)	0.840				
Insufficient	1.92 (1.03–3.57)	0.039				
FPG			−3.346 (−9.746 to 3.054)	−1.566 (−3.170 to 0.039)	0.254 (0.078 to 0.828)	0.022
<5.6 mmol/L	4.93 (1.14–21.35)	0.033				
≥5.6 mmol/L	1.19 (0.66–2.14)	0.569				

BMI, body mass index; FPG, fasting plasma glucose; HR, hazard ratio; CI, confidence interval; RERI, relative excessive risk due to interaction; AP, attributable proportion due to interaction; S, the synergy index.

*Serum 25(OH)D was categorized using 30 ng/ml and the reference group was serum 25(OH)D ≥30 ng/ml, the HRs were adjusted for age, gender, body mass index, district, education level, fasting plasma glucose, hypertension, dyslipidemia, smoking status, alcohol drinking, physical activity, and family history of diabetes.

^#^*P* for multiplicative interaction.

^*a*^Statistically significant with RERI > 0 and AP > 0 indicating additive interaction.

*^b^*The 95% CI of S was not available because the values of S were less than 0.

No evidence suggested significant additive or multiplicative interactions of serum 25(OH)D with residential district (Tables 4, 5).

### 3.5. Prediction value of serum 25(OH)D for type 2 diabetes

The category-free NRI of the model including serum 25(OH)D status improved by 0.205 (95% CI: 0.019–0.391, *P* = 0.034) for predicting T2D compared to the conventional risk model (Table 6). When serum 25(OH)D status was added to the conventional risk model, 19% more cases were correctly reclassified. Other analysis results, including the calibration, global goodness of fit, and discrimination of each model, can be found in Supplementary Table 4.

**TABLE 6:** Reclassification of type 2 diabetes risk after addition of serum 25(OH)D to conventional risk model.

	NRI (95% CI)*	*P* *	% of events correctly reclassified	*P*
Total	0.205 (0.019 to 0.391)	0.034	19%	0.039
**Age**				
<52 years	0.006 (−0.318 to 0.330)	0.973	−5%	0.746
≥52 years	0.349 (0.123 to 0.576)	0.004	26%	0.022
**Gender**				
Male	0.473 (0.199 to 0.747)	0.001	17%	0.216
Female	−0.017 (−0.270 to 0.235)	0.896	−21%	0.096
**BMI**				
<24 kg/m^2^	0.062 (−0.254 to 0.378)	0.701	0	1
≥24 kg/m^2^	0.249 (0.020 to 0.478)	0.041	30%	0.011
**Physical activity**				
Sufficient	0.146 (−0.292 to 0.584)	0.515	5%	0.827
Insufficient	0.293 (0.089 to 0.497)	0.006	25%	0.017
**FPG**				
<5.6 mmol/L	0.503 (0.171 to 0.835)	0.008	45%	0.016
≥5.6 mmol/L	0.076 (−0.145 to 0.297)	0.501	11%	0.329

BMI, body mass index; FPG, fasting plasma glucose; NRI, net reclassification index; CI, confidence interval.

*Serum 25(OH)D was categorized using median (25.415 ng/ml), conventional risk factors included age, sex, body mass index, district, education level, fasting plasma glucose, hypertension, dyslipidemia, smoking status, alcohol drinking, physical activity, and family history of diabetes.

The added predictive value of including serum 25(OH)D were more prominent in adults with age ≥52 years (NRI = 0.349, 95% CI: 0.123–0.576; *P* = 0.004), men (NRI = 0.473, 95% CI: 0.199–0.747; *P* = 0.001), adults with overweight/obesity (NRI = 0.249, 95% CI: 0.020–0.478; *P* = 0.041), adults with insufficient physical activity (NRI = 0.293, 95% CI: 0.089–0.497; *P* = 0.006) or with FPG <5.6 mmol/L at baseline (NRI = 0.503, 95% CI: 0.171–0.835; *P* = 0.008). Serum 25(OH)D correctly reclassified 26, 30, 25, and 45% more cases among those with age ≥52 years, with overweight/obesity, with insufficient physical activity, or with FPG <5.6 at baseline (Table 6).

## 4. Discussion

The relationship between serum 25(OH)D concentrations and T2D risk have attracted extensive attention during the past decades, and several previous studies, but not all, reported a significant inverse correlation between serum 25(OH)D and T2D risk. Several meta-analyses summarized these studies and concluded that low vitamin D levels might be associated with an increased risk of T2D (7–9). For instance, Song et al performed a meta-analysis on the data from 21 prospective studies and found that the summary risk ratio for T2D was 0.62 (95% CI: 0.54–0.70) when comparing the highest to the lowest category of 25(OH)D concentrations (9). The China Kadoorie Biobank (CKB) study indicated that a 10-ng/ml higher 25(OH)D concentration was associated with a 9% (95% CI: 0–18%) lower risk of incident diabetes after adjustment for age, gender, latitude, season, SBP, physical activity, and body fat percentage (34). While the Hong Kong Osteoporosis Study reported no association between serum vitamin D and the risk of incident diabetes in Hong Kong Chinese (35).

In the current study, the association between serum 25(OH)D concentrations and incident T2D was non-significant in an adult population from east-central China. The failure to detect significance might be due to the small sample size, short follow-up time, or high prevalence of vitamin D deficiency/insufficiency (77.21%). Vitamin D deficiency/insufficiency was prevalent in the Chinese population, especially among the elderly population and women (34, 36, 37). Due to the high prevalence of vitamin D deficiency/insufficiency, the sample size of the vitamin D sufficiency group was less than that of the vitamin D deficiency/insufficiency group, which might limit the ability to detect a significant association between vitamin D status and T2D risk. For example, the association of vitamin D deficiency/insufficiency with T2D risk was marginally significant after complete adjustments (adjusted HR = 1.66, 95% CI: 0.96–2.84; *P* = 0.068). Another possible explanation could be the study population who were at high risk for T2D (38). Vitamin D might be only beneficial in individuals with normal glucose tolerance because the development of T2D consisted of progressive insulin resistance, which was initially compensated by enhanced insulin secretion. At the onset of T2D, the β cell mass was reduced by 25–50% (39). When IFG is already present, the effect of vitamin D might be not strong enough to reverse the deterioration of glucose metabolism. In the current study, about half (51.71%) of the study participants had IFG at baseline. Stratified analysis showed that the effect of low serum 25(OH)D on T2D risk was significant in individuals with baseline FPG <5.6 mmol/L, but not among those with FPG ≥5.6 mmol/L at baseline. However, in a Swedish middle-aged population, high serum 25(OH)D predicted a lower risk of T2D in persons with prediabetes, but not among those with normal glucose tolerance (40). In participants with FPG <5.6 mmol/L, we found a non-linear inverse relationship between serum 25(OH)D and T2D with risk decreased rapidly when the serum 25(OH)D was between approximately 28.5 and 40 ng/ml, and when the serum 25(OH)D reached or exceeded approximately 40 ng/ml, the reducing trend of HR value tended to be flat. Previous studies reported that T2D risk increased significantly below the cutoff of 10 ng/ml (19), 16 ng/ml (21), and 18 ng/ml (20). Our results showed a significant multiplicative interaction between serum 25(OH)D and FPG on T2D risk, but the test of additive interaction was non-significant. The modification of baseline glucose metabolism status on the association between vitamin D and T2D risk deserves further investigation.

Stratified analyses also showed that the association between serum 25(OH)D and T2D risk was limited to men, adults with overweight/obesity, and individuals with insufficient physical activity. In agreement with our study, the MONICA 10 study showed that a significant inverse association of serum 25(OH)D concentrations with T2D risk was only evident among individuals with overweight/obesity (14). The Nurses’ Health Study also reported that the inverse association was stronger among women with overweight/obesity than women with normal BMI (13). However, results from the Copenhagen City Heart Study did not observe any effect modification by BMI (41). Interaction analysis between 25(OH)D and BMI was conducted in some studies. Most of them only did multiplicative interaction and reported negative results (12, 13, 41). For instance, no significant multiplicative interaction was observed between vitamin D biomarkers and BMI on diabetes incidence among older adults from the ESTHER study (18). But the MONICA 10 study reported significant multiplicative interaction between 25(OH)D and BMI as well as WC (14). Data from the NHANES indicated that there is no multiplicative or additive interaction between overweight/obesity and insufficient 25(OH)D on T2D (17). Our results suggested a potential additive interaction between vitamin D deficiency/insufficiency and overweight/obesity on T2D risk. And about 55.5% of the HR of being T2D was attributable to the interaction between these two factors. As more than half of the Chinese adult population is overweight/obesity and vitamin D deficiency/insufficiency is highly prevalent among persons with obesity (42, 43), our finding might have an important impact on public health.

As for gender and physical activity, two prospective studies reported that the inverse association of 25(OH)D with incident T2D did not differ by gender or physical activity, and no significant multiplicative interaction existed (14, 41). Our results suggested a potential additive interaction between serum 25(OH)D and male on T2D risk. Future large-scale studies are needed to confirm this gender difference. Our results also revealed an additive interaction between serum 25(OH)D and physical activity on T2D risk. The joint effects of low serum 25(OH)D and insufficient physical activity on T2D risk were greater than would be expected from the effects of the individual risk factor alone. This finding may have large public health significance because the burden of T2D among persons with insufficient physical activity may be decreased by making improvements in serum 25(OH)D concentrations. Solar exposure is a key determinant of serum 25(OH)D concentrations, as skin exposure to solar ultraviolet is a primary source of vitamin D. Therefore, increased outdoor physical activities during the daytime may lead to more solar exposure, which increases vitamin D synthesis. Thus, targeting lifestyle through sufficient outdoor physical activity may be the first option that will correct vitamin D deficiency/insufficiency and insufficient exercise. The mechanism underlying the additive interaction of serum 25(OH)D with physical activity on T2D risk is not clearly understood. In adults at high risk of T2D, moderate to vigorous physical activity was associated with lower concentrations of trimethylamine N-oxide (TMAO) (44), a novel gut-derived metabolite that was associated with insulin resistance and impaired glucose tolerance (45, 46). Animal studies showed vitamin D supplementation greatly reduced plasma TMAO levels in mice (47), and a cross-sectional study reported high TMAO concentrations were associated with vitamin D deficiency (48). It is plausible to speculate that higher TMAO due to insufficient physical activity may be aggravated by low serum 25(OH)D. Further studies are needed to clarify our results and explore the underlying mechanism.

In addition, we calculated the category-free NRI to measure the incremental predictive value of adding serum 25(OH)D status to conventional risk factors for the development of T2D. The addition of low serum 25(OH)D significantly improved the NRI for T2D by 0.205 in the total population. The improvement in the prediction of T2D, above conventional risk factors, demonstrated the potential of serum 25(OH)D as a biomarker for T2D risk. Stratified analyses indicated that the added predictive value of including serum 25(OH)D status was most evident among men, adults with older age, overweight/obesity, insufficient physical activity, or normal FPG. Few studies previously evaluated the impact of adding baseline serum 25(OH)D on the net reclassification of T2D risk beyond conventional risk factors. Researchers found that the addition of serum 25(OH)D to the Framingham Risk Score could improve coronary heart disease risk estimation in patients with essential hypertension or patients with T2D (22, 23). However, adding serum 25(OH)D in addition to established risk factors only marginally added prognostic value of mortality risk in patients undergoing coronary catheterization (49). Considering that vitamin D deficiency and insufficiency are easy to be diagnosed and cured, future studies are needed to evaluate the cost-benefit of adding serum 25(OH)D into the T2D risk estimation and then determine the feasibility of measuring serum 25(OH)D for T2D prevention, especially in high-risk populations.

To the best of our knowledge, the present study is the first to report an additive interaction between serum 25(OH)D concentrations and physical activity on T2D risk. Nevertheless, there are several limitations in the present study. First, the relatively small sample size, which limited our statistical power, and the short-term follow-up. The follow-up period might not be long enough to detect a modest effect and the association of serum 25(OH)D with T2D may be underpowered, especially within subgroup analysis. Second, we did not have data on oral glucose tolerance tests or HbA1c, which might lead to misclassification of the diagnosis of T2D. Third, a lack of information on serum 25(OH)D concentrations at follow-up might be a potential confounder and result in insufficient confirmation of our results. For instance, the Vitamin D and Type 2 Diabetes (D2d) study did not find a significant effect of vitamin D supplementation on diabetes risk, but a secondary analysis found that adults who maintained higher intratrial serum 25(OH)D levels during follow-up had a reduced risk of diabetes (50).

## 5. Conclusion

Our results suggest a non-linear reverse relationship between serum 25(OH)D and T2D risk among individuals with normal FPG at baseline, and a significant multiplicative interaction was detected between serum 25(OH)D and FPG on T2D risk. Significant additive interaction of serum 25(OH)D with male, overweight/obesity, and physical activity was observed. For T2D risk prediction, adding low serum 25(OH)D to the model with established risk factors might offer incremental predictive power. Our data are of significance to public health, and future studies are needed to confirm these findings and identify susceptible populations who can be greatly benefited from outdoor physical activity.

## Data availability statement

The original contributions presented in this study are included in the article/Supplementary material, further inquiries can be directed to the corresponding author.

## Ethics statement

The studies involving human participants were reviewed and approved by the Ethics Committee of Soochow University. The patients/participants provided their written informed consent to participate in this study.

## Author contributions

ZH, XZ, and ZZ: conceptualization, project administration, and funding acquisition. XZ, YM, and JW: methodology and data analyses. ZH and JZ: investigation and data collection. XZ, JL, and BL: manuscript writing and editing and final approval. All authors contributed to the article and approved the submitted version.
