# Supplementary material for: The modification of individual factors on association between serum 25(OH)D and incident type 2 diabetes: Results from a prospective cohort study
Source: Front Nutr. 2022 Dec 29;9:1077734. doi: 10.3389/fnut.2022.1077734 (PMC9835095; doi:10.3389/fnut.2022.1077734)
Supplement: Supplementary file 1 [file Data_Sheet_1.docx]

**Supplementary Figure S1.** Dose-response curves for the associations between serum 25(OH)D concentrations with risk of developing type 2 diabetes.

(A) among individuals with age < 52 years; (B) with female gender; (C) with normal BMI; (D) with sufficient physical activity; (E) with FBG ≥ 5.6 mmol/L at baseline. The solid line and dashed line represent the adjusted estimated HRs and 95% confidence intervals. Analyses were adjusted for age, gender, body mass index, district, education level, fasting plasma glucose, hypertension, dyslipidemia, smoking status, alcohol drinking, physical activity, and family history of diabetes.

**Supplementary Figure S2.** Dose-response curves for the associations between serum 25(OH)D concentrations with risk of developing type 2 diabetes among people living in different areas.

(A) among individuals living in rural areas; (B) among individuals living in urban areas. The solid line and dashed line represent the adjusted estimated HRs and 95% confidence intervals. Analyses were adjusted for age, gender, body mass index, education level, fasting plasma glucose, hypertension, dyslipidemia, smoking status, alcohol drinking, physical activity, and family history of diabetes.

# Supplementary Tables

Table S1. Hazard ratios (95% CIs) of type 2 diabetes by serum 25(OH)D concentrations among individuals with FPG less than 5.6 mmol/L

| 25(OH)D (ng/mL) | adjusted HR ^*^ | 95% CI ^*^ |
| --- | --- | --- |
| 20 | 1.00 (reference) | - |
| 22.5 | 0.97 | 0.79-1.19 |
| 28 | 0.54 | 0.29-1.01 |
| 28.5 | 0.50 | 0.25-0.99 |
| 29 | 0.46 | 0.22-0.97 |
| 30 | 0.38 | 0.16-0.92 |
| 31 | 0.32 | 0.12-0.88 |
| 32 | 0.26 | 0.08-0.84 |
| 33 | 0.21 | 0.06-0.80 |
| 34 | 0.17 | 0.04-0.76 |
| 35 | 0.14 | 0.03-0.73 |
| 36 | 0.12 | 0.02-0.70 |
| 37 | 0.09 | 0.01-0.68 |
| 38 | 0.08 | 0.01-0.63 |
| 39 | 0.06 | 0.01-0.63 |
| 40 | 0.05 | 0.00-0.60 |
| 41 | 0.04 | 0.00-0.58 |
| 42 | 0.03 | 0.00-0.56 |
| 43 | 0.03 | 0.00-0.54 |
| 44 | 0.02 | 0.00-0.52 |
| 45 | 0.02 | 0.00-0.50 |
| 46 | 0.01 | 0.00-0.49 |
| 47 | 0.01 | 0.00-0.47 |
| 48 | 0.01 | 0.00-0.45 |
| 49 | 0.01 | 0.00-0.44 |
| 50 | 0.01 | 0.00-0.42 |

FPG: fasting plasma glucose; HR: hazard ratio; CI: confidence interval;

*: adjusted for age, gender, body mass index, district, education level, fasting plasma glucose, hypertension, dyslipidemia, smoking status, alcohol drinking, physical activity, and family history of diabetes.

Table S2. Associations of baseline serum 25(OH)D concentrations (categorized using quartiles) with incident type 2 diabetes

| Subgroups | Q4 (≥ 29.55) | |  | Q3 (25.415-29.55) | |  | Q2(21.14-25.415) | |  | Q1(< 21.14) | |  | *P* for trend^#^ |
| --- | --- | --- | --- | --- | --- | --- | --- | --- | --- | --- | --- | --- | --- |
|  | HR (95% CI) ^*^ | *P* ^*^ | | HR (95% CI) ^*^ | *P* ^*^ | | HR (95% CI) ^*^ | *P* ^*^ | | HR (95% CI) ^*^ | *P* ^*^ | |  |
| Age |  |  | |  |  | |  |  | |  |  | |  |
| <52 years | 1.00 | - | | 1.38 (0.52-3.64) | 0.521 | | 1.48 (0.57-3.84) | 0.425 | | 0.85 (0.29-2.52) | 0.775 | | 0.760 |
| ≥52 years | 1.00 | - | | 1.09 (0.51-2.33) | 0.816 | | 1.47 (0.73-2.97) | 0.279 | | 1.81 (0.89-3.69) | 0.101 | | 0.062 |
| Gender |  |  | |  |  | |  |  | |  |  | |  |
| male | 1.00 | - | | 1.47 (0.63-3.43) | 0.378 | | 2.13 (0.96-4.71) | 0.063 | | 2.32 (0.99-5.41) | 0.052 | | 0.031 |
| female | 1.00 | - | | 0.89 (0.39-2.03) | 0.787 | | 1.08 (0.49-2.35) | 0.857 | | 0.89 (0.41-1.95) | 0.778 | | 0.855 |
| District |  |  | |  |  | |  |  | |  |  | |  |
| rural | 1.00 | - | | 1.26 (0.45-3.50) | 0.657 | | 1.35 (0.48-3.79) | 0.564 | | 1.65 (0.60-4.55) | 0.334 | | 0.332 |
| urban | 1.00 | - | | 1.15 (0.55-2.41) | 0.705 | | 1.60 (0.82-3.14) | 0.171 | | 1.28 (0.63-2.62) | 0.492 | | 0.396 |
| BMI |  |  | |  |  | |  |  | |  |  | |  |
| < 24 kg/m^2^ | 1.00 | - | | 0.83 (0.33-2.09) | 0.693 | | 0.99 (0.40-2.42) | 0.977 | | 1.02 (0.40-2.60) | 0.974 | | 0.903 |
| ≥ 24 kg/m^2^ | 1.00 | - | | 1.42 (0.63-3.20) | 0.405 | | 2.08 (0.96-4.49) | 0.064 | | 1.98 (0.90-4.35) | 0.091 | | 0.064 |
| Physical activity |  |  | |  |  | |  |  | |  |  | |  |
| sufficient | 1.00 | - | | 0.78 (0.21-2.87) | 0.705 | | 0.51 (0.12-2.15) | 0.355 | | 0.90 (0.22-3.62) | 0.881 | | 0.857 |
| insufficient | 1.00 | - | | 1.32 (0.67-2.60) | 0.428 | | 1.94 (1.04-3.63) | 0.038 | | 1.71 (0.89-3.30) | 0.107 | | 0.068 |
| FPG |  |  | |  |  | |  |  | |  |  | |  |
| < 5.6 mmol/L | 1.00 | - | | 3.81 (0.75-19.33) | 0.106 | | 8.38 (1.82-38.60) | 0.006 | | 4.68 (0.97-22.69) | 0.055 | | 0.051 |
| ≥ 5.6 mmol/L | 1.00 | - | | 0.90 (0.47-1.73) | 0.759 | | 0.95 (0.51-1.78) | 0.880 | | 1.08 (0.57-2.05) | 0.805 | | 0.740 |

BMI: body mass index, FPG: fasting plasma glucose, HR: hazard ratio, CI: confidence interval

*: adjusted for age, gender, body mass index, district, education level, fasting plasma glucose, hypertension, dyslipidemia, smoking status, alcohol drinking, physical activity, and family history of diabetes.

#: Test for trend based on the variable containing the median value for each group.

Table S3 Associations of serum 25(OH)D concentrations (categorized using quintiles) with incident type 2 diabetes

| Subgroups | Q5 (≥ 30.61) | Q4 (26.92-30.61) | Q3 (23.98-26.92) | Q2 (20.17-23.98) | Q1 (< 20.17) | *P* for trend^#^ |
| --- | --- | --- | --- | --- | --- | --- |
|  | HR (95% CI) ^*^ | HR (95% CI) ^*^ | HR (95% CI) ^*^ | HR (95% CI) ^*^ | HR (95% CI) ^*^ |  |
| Age |  |  |  |  |  |  |
| <52 years | 1.00 | 1.27 (0.42-3.90) | 1.66 (0.56-4.93) | 1.21 (0.39-3.80) | 0.68 (0.18-2.60) | 0.539 |
| ≥52 years | 1.00 | 0.92 (0.38-2.20) | 0.97 (0.41-2.26) | 1.65 (0.77-3.55) | 2.01 (0.94-4.28) | 0.018 |
| Gender |  |  |  |  |  |  |
| male | 1.00 | 1.14 (0.42-3.06) | 1.65 (0.66-4.15) | 2.57 (1.05-6.25) | 2.43 (0.94-6.25) | 0.015 |
| female | 1.00 | 0.95 (0.38-2.39) | 0.85 (0.34-2.13) | 0.92 (0.38-2.21) | 0.97 (0.42-2.28) | 0.999 |
| District |  |  |  |  |  |  |
| rural | 1.00 | 0.87 (0.26-2.92) | 1.32 (0.44-3.91) | 1.23 (0.40-3.78) | 1.83 (0.61-5.44) | 0.215 |
| urban | 1.00 | 1.21 (0.53-2.74) | 1.18 (0.52-2.69) | 1.65 (0.77-3.56) | 1.41 (0.64-3.13) | 0.280 |
| BMI |  |  |  |  |  |  |
| < 24 kg/m^2^ | 1.00 | 0.70 (0.26-1.90) | 0.55 (0.18-1.66) | 1.20 (0.46-3.09) | 0.92 (0.33-2.53) | 0.848 |
| ≥ 24 kg/m^2^ | 1.00 | 1.50 (0.56-4.04) | 1.95 (0.76-4.99) | 2.04 (0.81-5.15) | 2.61 (1.03-6.62) | 0.026 |
| Physical activity |  |  |  |  |  |  |
| sufficient | 1.00 | 0.86 (0.20-3.60) | 0.69 (0.14-3.35) | 0.43 (0.08-2.33) | 1.10 (0.25-4.84) | 0.975 |
| insufficient | 1.00 | 1.15 (0.53-2.50) | 1.38 (0.66-2.87) | 2.03 (1.01-4.10) | 1.81 (0.88-3.76) | 0.034 |
| FPG |  |  |  |  |  |  |
| < 5.6 mmol/L | 1.00 | 2.47 (0.44-13.78) | 5.14 (1.06-25.08) | 5.96 (1.22-29.03) | 3.33 (0.66-16.90) | 0.130 |
| ≥ 5.6 mmol/L | 1.00 | 0.84 (0.40-1.77) | 0.79 (0.37-1.67) | 1.02 (0.51-2.03) | 1.30 (0.64-2.61) | 0.319 |

BMI: body mass index, FPG: fasting plasma glucose, HR: hazard ratio, CI: confidence interval

*: adjusted for age, gender, body mass index, district, education level, fasting plasma glucose, hypertension, dyslipidemia, smoking status, alcohol drinking, physical activity, and family history of diabetes.

#: Test for trend based on the variable containing the median value for each group.

Table S4. Reclassification of type 2 diabetes after addition of serum 25(OH)D

|  | Baseline model^*^ | Baseline model + 25(OH)D |
| --- | --- | --- |
| Model calibration, Gronnesby and Borgan χ^2^ (*P* value) | 0.835 (0.3607) | 0.668 (0.4136) |
| Model global fit, AIC | 1645.248 | 1644.793 |
| Model global fit, BIC | 1723.132 | 1728.241 |
| Likelihood ration test |  | *P*=0.117 |
| Harrell's C index | 0.768 | 0.771 |
| IDI |  | 0.0018 (-0.0016-0.0052); *P*=0.309 |
| Relative IDI (%) |  | 2.48% |

Gronnesby and Borgan χ^2^ statistic was used to evaluate the calibration of the models.

AIC and BIC: Akaike information criteria and Bayesian information criteria; evaluating the global goodness of fit.

Likelihood ratio test was used for comparing two models.

Harrell’s C index: measure of discrimination of events versus nonevents.

IDI: integrated discrimination improvement; assessing the capacity of an added biomarker to an existing predicting model

*: variables used in the baseline model included age, sex, body mass index, district, education level, fasting plasma glucose, hypertension, dyslipidemia, smoking status, alcohol drinking, physical activity, and family history of diabetes.
