# Supplementary figures and images for: The modification of individual factors on association between serum 25(OH)D and incident type 2 diabetes: Results from a prospective cohort study
Source: Front Nutr. 2022 Dec 29;9:1077734. doi: 10.3389/fnut.2022.1077734 (PMC9835095; doi:10.3389/fnut.2022.1077734)

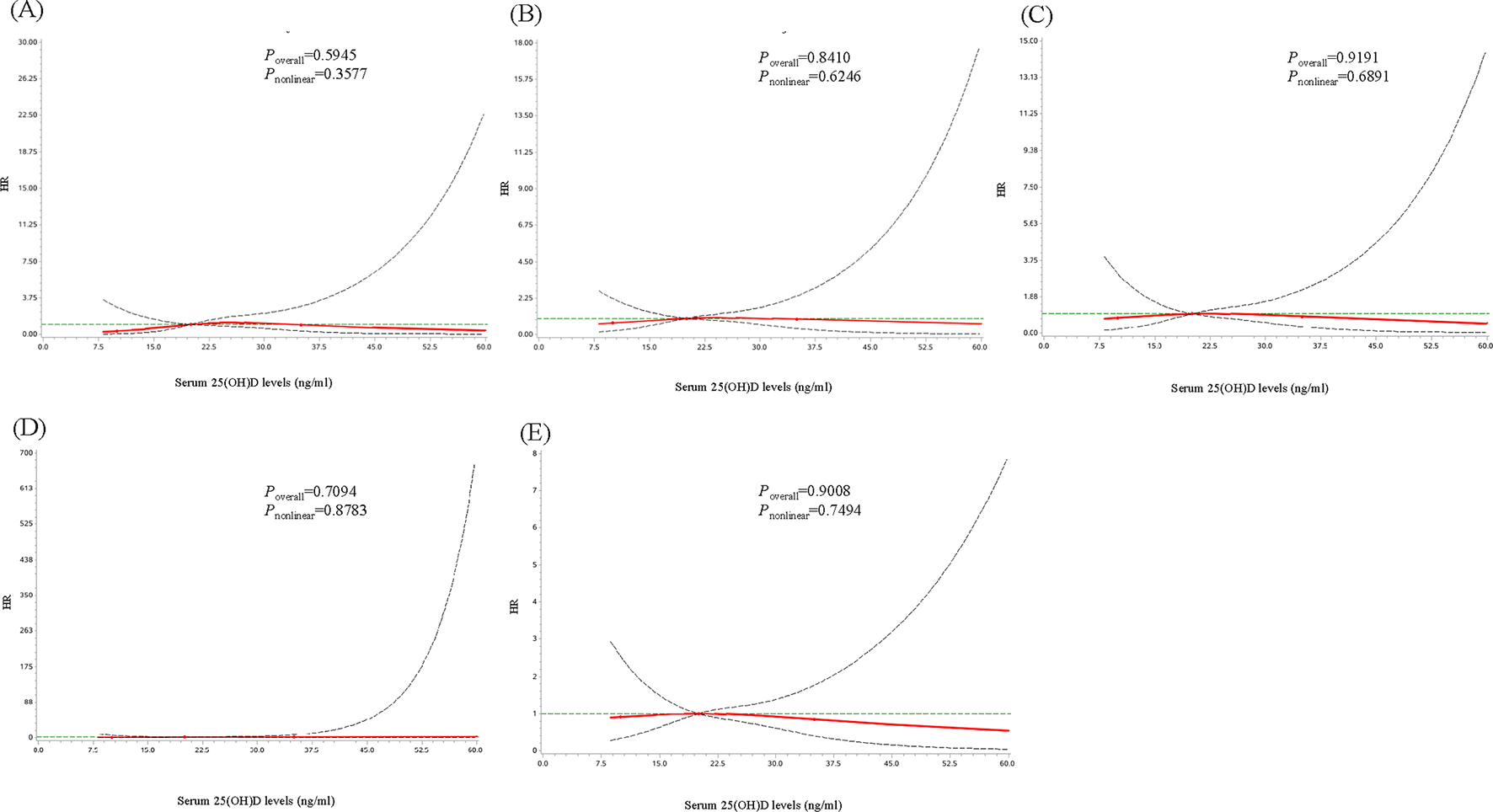

Supplement: Supplementary file 2 [file Image_1.TIF]

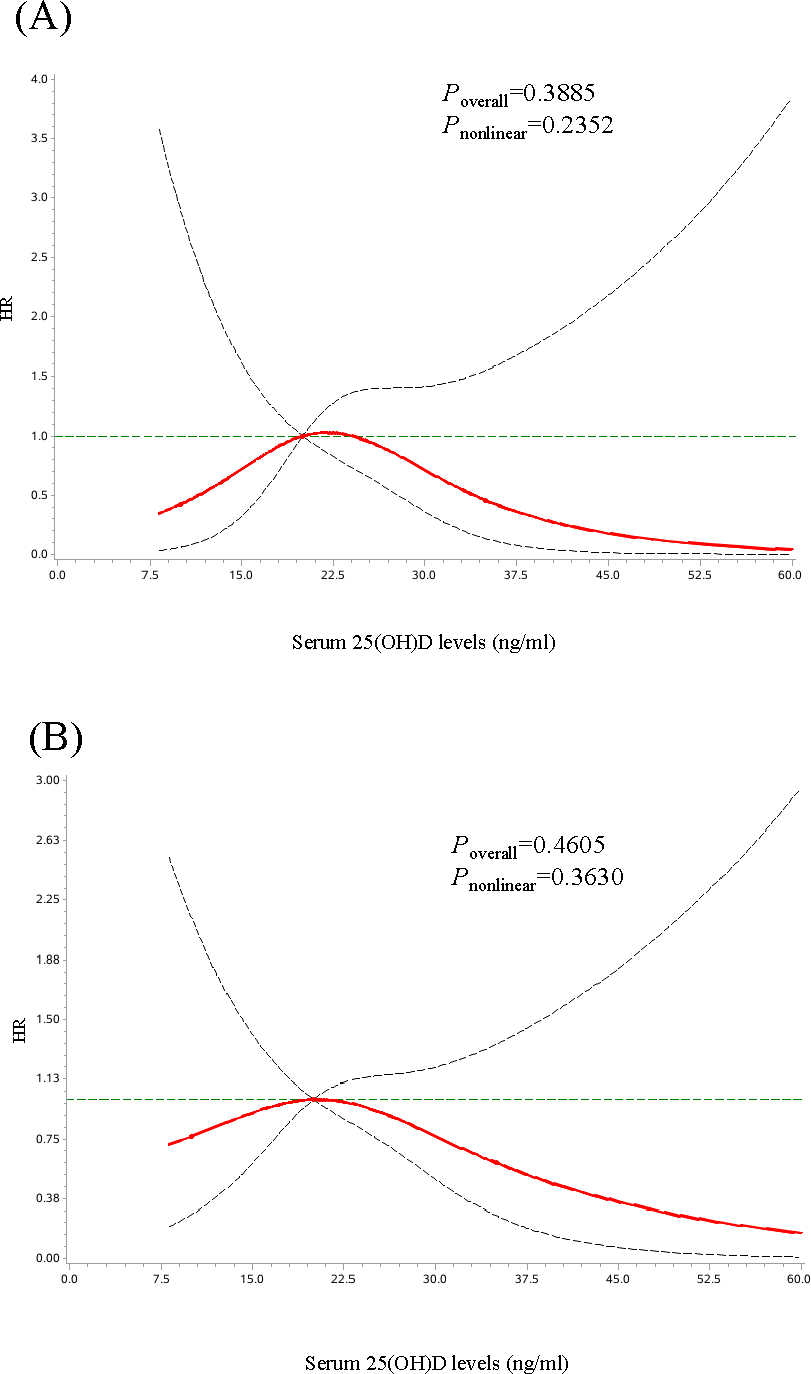

Supplement: Supplementary file 3 [file Image_2.TIF]
